# Reporter gene-based optoacoustic imaging of *E. coli* targeted colon cancer in vivo

**DOI:** 10.1038/s41598-021-04047-4

**Published:** 2021-12-24

**Authors:** Misun Yun, Sung-Hwan You, Vu Hong Nguyen, Jaya Prakash, Sarah Glasl, Vipul Gujrati, Hyon E. Choy, Andre C. Stiel, Jung-Joon Min, Vasilis Ntziachristos

**Affiliations:** 1 Institute of Biological and Medical Imaging, Helmholtz Zentrum München GmbH, 85764 Neuherberg, Germany; 2 Department of Nuclear Medicine, Chonnam National University Medical School, Gwangju, Republic of Korea; 3 Department of Experimental Therapeutics, Beckman Research Institute of City of Hope, Duarte, CA USA; 4 Department of Instrumentation and Applied Physics, Indian Institute of Science, C. V. Raman Avenue, Bengaluru, 560 012 India; 5 Department of Microbiology, Chonnam National University Medical School, Gwangju, Republic of Korea; 6 Chair of Biological Imaging at the Center for Translational Cancer Research (TranslaTUM), School of Medicine, Technical University of Munich, 81675 Munich, Germany

**Keywords:** Imaging, Optical imaging, Ultrasound, Biological techniques, Cancer imaging, Cancer imaging

## Abstract

Bacteria-mediated cancer-targeted therapy is a novel experimental strategy for the treatment of cancers. Bacteria can be engineered to overcome a major challenge of existing therapeutics by differentiating between malignant and healthy tissue. A prerequisite for further development and study of engineered bacteria is a suitable imaging concept which allows bacterial visualization in tissue and monitoring bacterial targeting and proliferation. Optoacoustics (OA) is an evolving technology allowing whole-tumor imaging and thereby direct observation of bacterial colonization in tumor regions. However, bacterial detection using OA is currently hampered by the lack of endogenous contrast or suitable transgene fluorescent labels. Here, we demonstrate improved visualization of cancer-targeting bacteria using OA imaging and *E. coli* engineered to express tyrosinase, which uses L-tyrosine as the substrate to produce the strong optoacoustic probe melanin in the tumor microenvironment. Tumors of animals injected with tyrosinase-expressing *E. coli* showed strong melanin signals, allowing to resolve bacterial growth in the tumor over time using multispectral OA tomography (MSOT). MSOT imaging of melanin accumulation in tumors was confirmed by melanin and *E. coli* staining. Our results demonstrate that using tyrosinase-expressing *E. coli* enables non-invasive, longitudinal monitoring of bacterial targeting and proliferation in cancer using MSOT.

## Introduction

Tumor-targeting bacteria are becoming increasingly more attractive as a theranostic platform. They can be genetically engineered to localize tumors and trigger a therapeutic effect while carrying detectable labels for additional diagnostic imaging^1–3^. Current imaging strategies for the development of bacteria-mediated cancer therapy in small animals rely largely on optical methods like bioluminescence and fluorescence for visualization^4,5^. These optical approaches are incapable of providing additional anatomical or physiological information, nor can they offer the resolution necessary to non-invasively analyze the details of bacterial cancer targeting and efficacy^6,7^. Radiological methods like magnetic resonance imaging (MRI) or positron emission imaging (PET) have also been explored, but these techniques suffer from limitations in sensitivity and resolution, respectively. Furthermore, the high cost associated with these methods and the use of radioisotopes (PET) makes them unsuitable for routine applications. Altogether, these challenges limit the possibilities for detailed studies of novel theranostic agents, their mechanisms of action, and the responses of tumors and their hosts to treatment.

Optoacoustic (OA) imaging, based on optical excitation and ultrasound detection, represents a promising solution to the above limitations, as it is a powerful molecular imaging modality owing to the unique combination of high spatio-temporal resolution, deep penetration and spectrally-enriched contrast^7^. This method therefore offers many possibilities for labeling strategies. Moreover, because the detection of ultrasound is not limited by photon scattering, it enables scalable high-resolution imaging deep in the tissue (20–200 µm)^7^. In particular, multispectral optoacoustic tomography (MSOT) was proven to be effective for preclinical imaging of tumors in small animals and has been implemented in a handheld clinical system for tumor assessment^7–9^. MSOT is regularly used to detect contrast from endogenous absorbers such as hemoglobin, lipids and water, or from exogenous absorbers such as dyes or nanoparticles^9^. Labeling bacteria with transgene fluorescent or chromoproteins for detection in OA has however thus far proven unsuccessful due to weak acoustic signals and often poor photostability, limiting sensitivity and observation time^10^; photo-switching chromoproteins provide a promising alternative but their development and use are still in its infancy^11^. In contrast, a particularly strong OA signal can be generated by expressing the gene encoding tyrosinase, which is the rate-limiting enzyme in the production of melanin, a strong contrast agent for OA imaging^12,13^.

In the presence of the substrate l-tyrosine, cells produce melanin which in turn allows their detection with great sensitivity by MSOT^12,14,15^. Here, we use MSOT to monitor bacterial targeting, infiltration and proliferation specifically in the tumor microenvironment based on *E. coli* that express the transgene for tyrosinase. After injection into mouse tumors, the enzyme is secreted out of the bacterial cells and converts l-tyrosine in the surrounding environment into melanin. We were able to track accumulation of the bacteria in the tumor, which was also confirmed using histology via labelling of melanin and bacteria. This study suggests that tyrosinase-expressing *E. coli* may be an effective optoacoustic imaging agent with potential utility in bacterial therapy. To our knowledge, this is the first report of optoacoustic imaging of melanin contrast in tumor-targeting bacteria in-vivo.

## Results

### Characterization of melanin production in vitro

**Figure 1:**
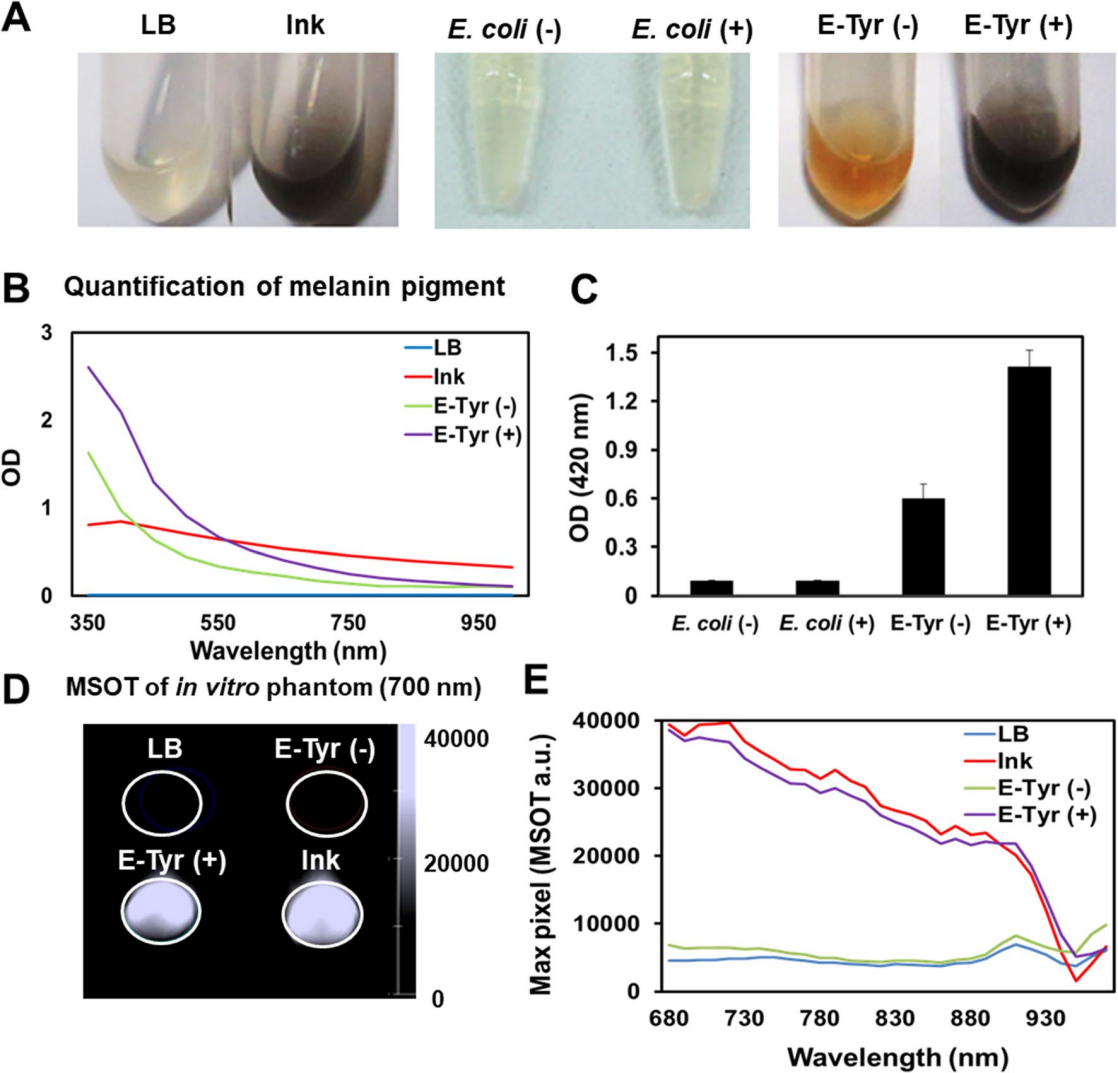
Production of melanin by E-Tyr in vitro. (**A**) Melanin production in vitro*.* Tyrosinase-expressing *E. coli* (E-Tyr) were grown overnight in the presence or absence of L-tyrosine supplementation. (left) LB (growth medium) and ink control, (middle) supernatant of *E. coli* with the tyrosinase-expression vector (control bacteria) cultured with (*E. coli*(+)) or without (*E. coli*(−)) L-tyrosine supplementation, and (right) supernatant of *E. coli* carrying the tyrosinase-expression vector cultured with (E-Tyr(+)) or without (E-Tyr(−)) l-tyrosine supplementation. (**B**) Optical density measurements of LB, ink, E-Tyr (−) and E-Tyr (+) over the spectrum from 350 to 1000 nm. (**C**) Quantification of melanin pigment shown in (**A**), *E. coli* (−), *E. coli* (+), E-Tyr (−) and E-Tyr (+), using a spectrophotometer at 420 nm wavelength. (**D**) Optoacoustic in vitro phantom imaging of (**C**) with an optoacoustic image spectrum peaked at 700 nm. (**E**) Optoacoustic spectra for E-Tyr with or without L-tyrosine supplementation, ink and LB media, measured between 680 and 970 nm.

Towards testing the functionality of melanin production in bacteria, we transfected *E. coli* (MG1655) with a plasmid encoding tyrosinase and its “caddie” protein, which together form the active complex. Cells cultured overnight in LB-medium showed a clear presence of black pigment only when growing in the presence of L-tyrosine (Fig. 1A), confirming that engineered bacteria (E-Tyr) only produced substantial melanin in the presence of tyrosine substrate. The negligible amount of melanin production in the culture without L-tyrosine likely reflects the presence of this amino acid in the culture medium composition. After centrifuging the bacteria, melanin was visually detectable only in the supernatant but not the pellet, confirming the secretion of functional tyrosinase^14,16^. Since the enzyme encounters l-tyrosine only after secretion from the bacterial cell^16–19^, melanin production is generated exclusively in the surrounding medium, although the exact mechanism of melanin precursors release is not known^16,19^. Figure 1B shows the characteristically strong melanin absorption below 450 nm in E-Tyr cultures in the presence of added l-tyrosine and, to a lesser extent, in its absence^19–21^. Figure 1C quantifies the results from Fig. 1A and shows the levels of melanin produced by the bacteria based on spectrophotometry at 420 nm. After demonstrating production of melanin by E-Tyr, we examined whether the pigment could be detected using MSOT. Tubes containing supernatants from E-Tyr cultures with or without added l-tyrosine were placed inside agar phantoms. Figure 1D shows that melanin produced by E-Tyr cultured in the presence of l-tyrosine generated a strong optoacoustic signal. Only a weak MSOT signal was obtained with the supernatant of E-Tyr cultured in the absence of l-tyrosine. Figure 1E demonstrates that the melanin produced by E-Tyr exhibits an MSOT spectrum similar to the spectrum shown previously for melanin-producing cells^13^. Importantly, in contrast to the control culture without L-tyrosine and pure LB-medium, we see a generally increased signal for E-Tyr and the characteristic decay of the signal over the whole recorded spectra. Notably, there is a larger deviation between the ink and melanin-producing *E. coli* compared to the controls when using MSOT than when using a spectrometer, particularly visible in the pronounced drop around 900 nm. This deviation might be due to several factors like non-linear variations in fluence distribution across different wavelengths, fluctuations in laser energy at different wavelengths, the spatial impulse response of the transducer, temperature-dependent thermal conversion factors (Grüneisen parameter) and optical scattering^22–25^. In summary, these results indicate that tyrosinase-expressing *E. coli* can produce melanin that can be detected by MSOT in vitro.

### MSOT imaging of CT26 tumors in vivo

**Figure 2:**
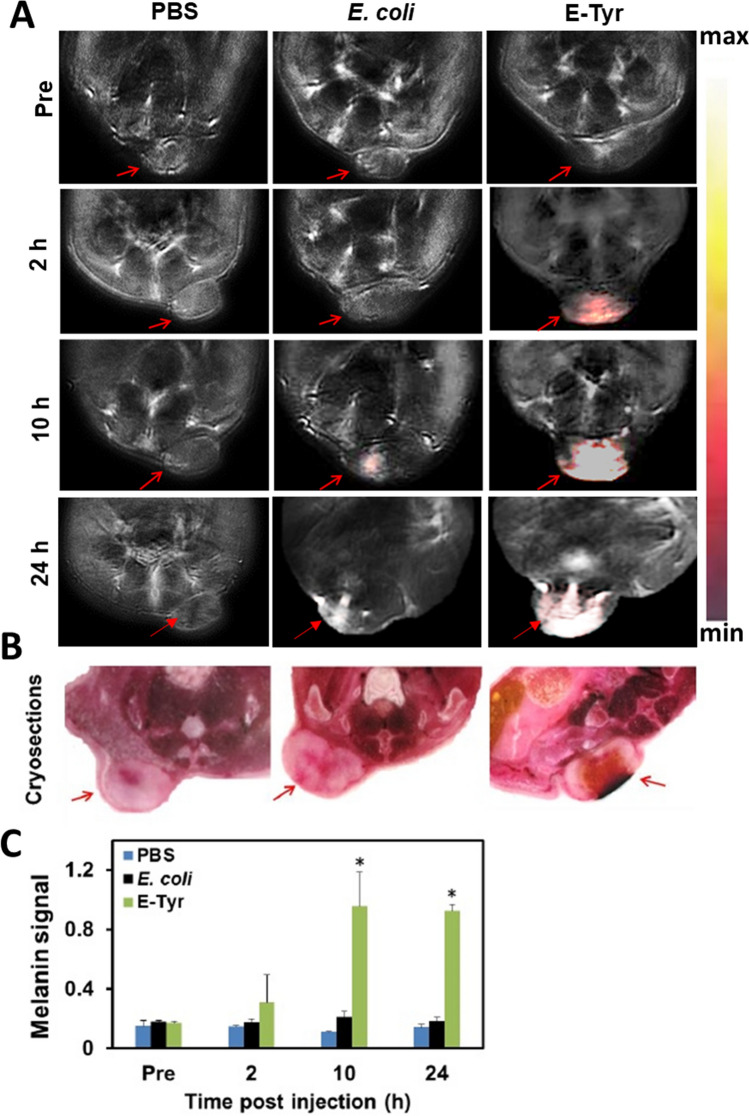
MSOT imaging of CT26 tumors in mice after injection with E-Tyr. (**A**) MSOT images of melanin signal in CT26 tumor bearing live mice (n = 6/group) with the indicated time after E-Tyr (1 × 10^8^ cfu) injection. Unmixed melanin overlaid on anatomy. The tumor area is indicated by a red arrow (left; PBS injected control mice, middle; *E. coli* injected mice, right; E-Tyr injected mice) (**B**) Cryo-slice images of (**A**) indicated tumor by red arrow after MSOT imaging. (**C**) Quantitative optoacoustic signal for melanin of tumors at indicated times for PBS, *E. coli* and E-Tyr injected mice. (*P < 0.05).

We next set out to investigate whether E-Tyr-induced melanin could be similarly detected in vivo. Mice bearing CT26 tumors were intravenously injected with *E. coli* with or without the tyrosinase transgene and subsequently anesthetized and imaged using MSOT. Figure 2A shows that the melanin signal in the tumor was much stronger in the group injected with E-Tyr than in the control groups. For all the cases considered here, the unmixing was performed with the melanin chromophore included along with oxyhemoglobin and deoxyhemoglobin chromophores; hence it is expected that some unmixing errors (corresponding to melanin chromophore) would start appearing since melanin was used in the unmixing process. Therefore, we obtained signals corresponding to melanin chromophore in the E. coli 10 h post-injection image (false positive). Moreover, the signal unmixed using E coli was about 9 times weaker compared to E-Tyr, indicating the presence of melanin in the E Tyr treatment group as opposed to E coli. Figure 2B shows a comparison of unstained tumor cryoslices approximately matched to the positions imaged in MSOT. Black coloring can only be seen in the group injected with E-Tyr, suggesting the presence of abundant melanin pigment. Figure 2C shows the quantification of the melanin signal in the tumor before and after bacterial injection. Further, the SO_2_ distribution obtained from these different mice is shown in the supplementary section (Fig. S2).

### Correlation between tumor optoacoustic imaging and the distribution of melanin in tumor cryosections

**Figure 3:**
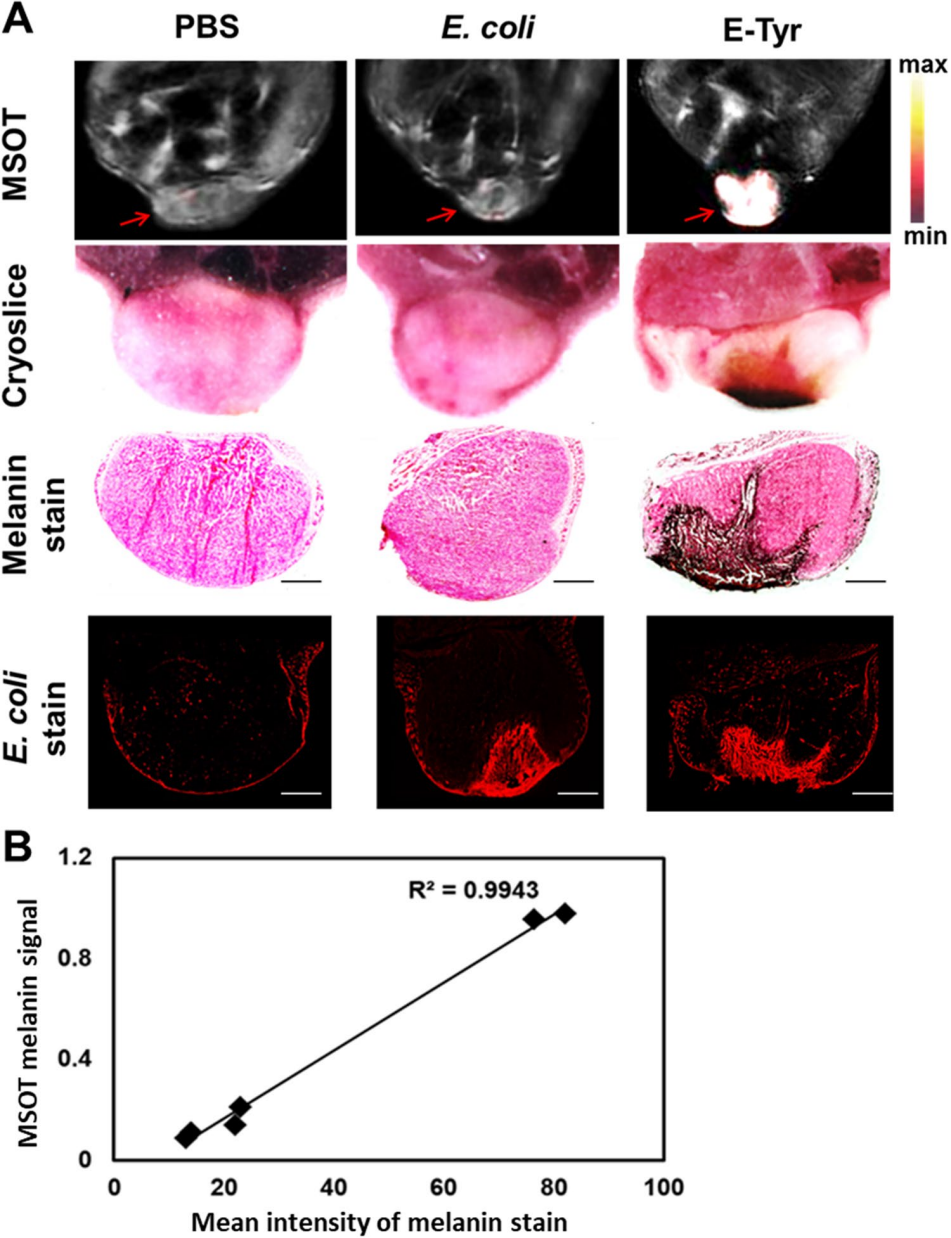
Correlation between tumor optoacoustic melanin signal in vivo and the distribution of melanin in tumor cryo sections. (**A**) MSOT images of melanin signal using CT26 tumor-bearing live mice 24 h after E-Tyr injection (Top). Cyro-slice image of tumors. Tumors were assessed by specific melanin staining (darkbrown, middle) using the Fontana-Masson stain and *E. coli* stain (red fluorescence, bottom) with a specific *E. coli* antibody. High magnification (× 100) images of melanin and *E. coli* stains were obtained using the software ZENblue (CarlZeiss). (**B**) Correlation of the melanin signal of MSOT (24 h) and the mean intensity of melanin stain by the Pearson correlation method (R^2^ = 0.9943, n = 6).

To further confirm the above findings, we performed staining’s to specifically detect the presence of melanin and *E. coli*. Figure 3A (top) shows a Fontana-Masson stain, which predominantly marks melanin. The signals can be found in similar regions as the MSOT signals and the *E. coli* distribution based on antibody staining (bottom), suggesting that the signal observed in MSOT stems from melanin. Even though abundant bacteria are visible in the tumor in the control *E. coli* group, no appreciable melanin is visible, supporting that our MSOT images selectively detect melanin in tumors. Figure 3B shows the correlation between the mean intensity of Fontana-Masson staining against melanin and the MSOT signal at 24 h after injection. Three additional tumor-bearing mice per group were visually monitored until 72 h for melanin production (Fig. S1), with only the tumors corresponding to E-Tyr showing black coloring, indicating that melanin was produced in the tumor region. This demonstrates that MSOT can routinely detect melanin produced by tyrosinase-expressing bacteria deep in the tumor. Sufficient l-tyrosine is seemingly present in the tumor microenvironment for the secreted tyrosinase to produce melanin, and accumulation of E-Tyr was already detectable by 10 h after systemic injection in our tumor-bearing mice. Altogether, these findings support the potential of E-Tyr for MSOT-based theranostics. We further investigated the bacterial colonization in the vital organs and tumor of CT26 tumor-bearing Balb/c mice (Fig. S3). High bacterial counts were observed in the tumor of immunocompetent mice. No long-term mortality was recorded after bacterial injection of the bacteria and we furthermore confirmed absence of any damage in normal tissues (heart, lung, spleen liver) in bacteria-treated Balb/c athymic nu-/nu mice compared with PBS-treated mice (Fig. S4).

## Discussion

The challenges faced by current antitumor chemotherapeutics, such as high toxic side effects, inability to treat poorly oxygenated deep tumor tissue, and drug resistance, have prompted the development of alternative approaches^26^. Live tumor-targeting bacteria have generated interest due to their versatile capabilities for targeting and suppressing primary and metastatic tumors^2,27^. Bacteria can be made to preferentially accumulate within tumors, where they can deliver cancer-specific payloads, attract immune cells or initiate antitumor immune responses^28^. Furthermore, bacteria can be programmed via sophisticated genetic engineering to produce and deliver anticancer agents based on clinical needs. In addition to therapeutic payload delivery, bacteria can be engineered to diagnose and visualize tumors^1,5,29–31^. The ability to noninvasively monitor bacterial tumor targeting, as well as tumor interaction and proliferation, is uniquely suited for accurate diagnosis and therapeutic response predictions.

Here we introduce the engineered canonical *E. coli* strain MG1655 that expresses tyrosinase to produce the biopolymer melanin, which is a strong absorber of light and generator of strong acoustic signals. One major challenge of melanin identification in tumors stems from the overlap in the absorption spectra of melanin and deoxyhemoglobin. The spectral signature of deoxyhemoglobin and melanin are not orthogonal to each other and both show a decrease in absorption with increasing wavelength^12^. Necrotic tumors are characterized by an increased deoxyhemoglobin footprint. However, the optoacoustic signal generated by E-Tyr was very strong and showed the typical melanin signature, while the crest and trough hallmark signature of deoxyhemoglobin in the 730–780 nm region was insubstantial, indicating overexpression of melanin (Fig. 1E). We also observed that the optoacoustic signal decreased sharply in the 800–900 nm wavelength range in the E-Tyr group, while it remained constant in the control groups (LB and E-Tyr (–)), which indicates the presence of melanin in the E-Tyr group and deoxyhemoglobin in the control group, as observed previously^12^. This is the first demonstration of revealing a tumor by optoacoustic imaging using tumor-targeting *E. coli* that promote melanin production, and this strategy represents a promising potential candidate as a theranostic agent in the near future^28,32,33^. Lastly, development of multispectral optoacoustic endoscopes is underway, which could potentially enable the clinical translation of theranostic approaches in those organs which exceed the maximum penetration depth of transcutaneous MSOT.

## Summary

Engineered bacteria have the unique capability of targeting tumors when systemically administered, resulting in high levels of local replication in the tumor microenvironment and within cancer cells. Both pre-clinical and clinical investigations involving bacteria as imaging or therapeutic vectors require an efficient means of monitoring bacterial trafficking in real-time. However, the lack of a suitable transgene contrast agent has prevented the application of bacteria in optoacoustic imaging of cancer. Our approach of using tyrosinase-expressing E. coli enables non-invasive, longitudinal monitoring of bacterial targeting and proliferation in cancer using multispectral optoacoustic tomography. Our results clearly indicate a potential to adapt similar approaches of transgene delivery and bacterial production for imaging or therapeutics of deeply seated tumors, which will considerably enhance our understanding of the complexities of the disease in vivo.

## Methods

All procedures involving animal experiments were approved by the Government of Upper Bavaria and carried out in accordance with institutional guidelines and in compliance with the ARRIVE guidelines.

### Plasmid construction

ORF378 and the tyrC in the mel operon were amplified from *Streptomyces castaneoglobisporus* by PCR amplification using two primers: F (5’- CGA AAG CTT TGA TCA CGT CAG TTT TCG CAC GTG -3’), R (CGA TCT AGA TGA TCA GAC GTC GAA GGT GTA GTG C). PCR fragments were digested by HindIII and XbaI restriction enzymes and ligated into a pUC19 vector, generating pUM378.3. This construct was transformed into *E. coli* K-12 MG1655 and maintained under ampicillin selection.

### Bacterial culture

The wild-type *E. coli* K-12 strain (MG1655) was used in the present study^5,30^. Tyrosinase-expressing *E. coli* (E-Tyr) were routinely grown overnight in Luria–Bertani (LB) medium in a shaking incubator at 37 °C. For injection, E-Tyr culture grown overnight was diluted 1:100 in LB and incubated at 37 °C for 5 h in order to reach the early exponential phase of bacterial growth. Bacteria were harvested and washed once with PBS and measured.

### Optical density measurements and calculations

For optical density measurements, Tyr-E samples harvested from supernatants with/without L-tyrosine by centrifugation (Sigma-Aldrich Chemicals Co. St. Louis, USA) were measured with a MOI of 1 [an optical density of 1; OD600]. The tyrosine was pre-dissolved and then added to the LB liquid. The supernatant was measured in 10-nm increments from 350 to 1000 nm on a SpectraMax M5 reader (Molecular Devices). Relative optical density, compared with culture media and Indian ink, were calculated by dividing the absorbance data from each wavelength.

### Measurement of optoacoustic spectra and MSOT imaging in vitro

The supernatants (300 μL of the same suspension from which optical density measurements were obtained) were filled into an agar phantom (3% agarose in PBS) with four cylindrical holes of 3 mm in diameter. The MSOT data acquisition was performed with transversal plane imaging at a single position, located approximately in the middle of the phantom^12^. Imaging was performed at a 680–900 nm wavelength range. The phantom images were reconstructed at a wavelength of 700 nm. Spectra are shown as the maximum value at each wavelength.

### Cell lines and animals for MSOT imaging in vivo

Six-week-old female BALB/c-nu/nu mice were purchased from Envigo (Germany). CT26 cells (mouse colon carcinoma) were grown as monolayers in DMEM medium (Invitrogen) supplemented with 10% (v/v) fetal bovine serum (Invitrogen) and 1% Penicillin–Streptomycin antibiotics (Invitrogen). Tumor-bearing nude mice were generated by subcutaneous injection with 1 × 10^6^ cells suspended in 50 μL PBS in the right flank. After approximately 10 days, the animals bearing tumor sizes of approximately 80 mm^3^ were used for experiments. These were injected intravenously in the tail vein with sterile PBS, 1 × 10^8^ cfu bacteria resuspended in 100 µL sterile PBS. MSOT imaging was performed over the period of 24 h post-injection. The effect of E-Tyr on survival was monitored for a month in immunocompromised Balb/c athymic nu-/nu- mice that are injected with 10^8^ cfu of E-Tyr/mouse through intravenous injection (n = 3 per group). Additional safety study was done in CT26 tumor bearing competent mice. Animals received 10^8^ cfu of E-Tyr through intravenous injection and vital organs including heart, lung, liver, spleen, and tumor tissues were harvested for viable bacterial count and immunostaining^27^. Sample sizes were chosen based on guidance from the literature. Investigators were not blinded to the identity of groups. For all animal studies, animals of the same gender, age, and genetic background were randomized for grouping.

### Multispectral optoacoustic imaging

Optoacoustic imaging was performed using a real-time whole-body mouse imaging scanner, MSOT In Vision 256-TF (iThera-Medical GmbH, Munich, Germany)^12^. Briefly, the system utilizes a cylindrically focused 256-element transducer array at 5 MHz central frequency covering an angle of 270°. The system acquires cross-sectional (transverse) images of the animal. The animals are placed onto a thin clear polyethylene membrane. The membrane separates the animals from a water bath, which is maintained at 34 °C and is used for acoustic coupling and maintaining animal temperature while imaging. Image acquisition speed is at 10 Hz. The data is acquired using a multi-channel DAQ with a sampling rate of 40 megasamples per second. Imaging was performed at wavelengths from 680 to 900 nm with a stepsize of 5 nm, and at 20 consecutive slices with a step size of 0.5 mm. The acquired data was filtered with a Chebyshev filter with a bandwidth as 150 kHz–8 MHz. Image reconstruction was performed using a non-negativity constraint imposed during inversion and with Tikhonov regularization using a least-squares QR (LSQR) solver with 100 iterations. Further, the speed of sound was set to 1540 m/s in all these reconstructions. This work involves unmixing multiple chromophores like hemoglobin, deoxyhemoglobin, and melanin. Furthermore, the unmixing problem is highly nonlinear due to the fluence effects, the spatial impulse response of the transducer and the temperature-dependent thermal conversion factors (Grüneisen parameter). However, we have ignored these effects and assumed the problem to be linear, hence we have performed linear unmixing of these different chromophores. Kindly note that these are intrinsic chromophores and the illumination energy used is within ANSI limits to avoid any absorption saturation effects^34^. Lastly, since the problem is highly non-linear, it is recommended to use more wavelengths compared to the number of chromophores being unmixed, making the linear unmixing problem overdetermined and less ill-posed. We therefore used 44 wavelengths (leading to more data for unmixing).

### Histology and microscopy

After completion of imaging, the mice were euthanized with a lethal dose of ketamine/xylazine and frozen to − 20 °C. The upper torso including the tumor was embedded in O.C.T. (TissueTec). The embedded tumor tissue was sliced along the axial plane every 50 µm in a modified Leica cryotome combined with a CCD camera to capture RGB color images from the surface of the bulked remaining sample. For melanin pigment staining, the sliced tumor was fixed with 4% PFA, stained using the Fontana-Masson stain kit (Abcam), as per directions of the manufacturer. Briefly, tumor slices were incubated with ammonical silver solution until it became brown (around 30 min) indicating melanin staining. Next, the slices were incubated with gold chloride and sodium thiosulfate solution before staining with nuclear fast red solution. Samples were rinsed throughout as per the manufacturer’s protocol using distilled water and finally mounted using mounting media. For bacterial detection, the tissue sections were incubated with the primary antibody for *E. coli* (Dako B-357) for 2 h at RT. After washing with PBS, the secondary antibodies conjugated with mCherry594 were applied and continuously incubated for 1 h at RT. Microscopy of the tissue sections was performed with an Olympus IX81 confocal microscope (Zeiss Axio Imager M2 microscope).

### Administration of bacteria in tumor-bearing mice

The mice bearing approximately 80 mm^3^ s*.c.* tumors were injected intravenously with sterile PBS and 1 × 10^8^ cfu bacteria resuspended in 100 µL sterile PBS. Tumor sizes were measured individually with a caliper every day.

### Statistical analysis

All data are presented as the mean ± SD. A statistical analysis was performed using Student’s t-test, with a p-value of less than 0.05 as a cutoff, in GraphPad Prism (GraphPad Software Inc.). Correlations between melanin imaging signals and intensity of melanin stain were analyzed by Pearson’s correlation.

## Supplementary Information

Supplementary Information.

## Data Availability

All data presented in the paper are available from the authors upon reasonable request.
