# Supplementary material for: Reporter gene-based optoacoustic imaging of E. coli targeted colon cancer in vivo
Source: Sci Rep. 2021 Dec 24;11:24430. doi: 10.1038/s41598-021-04047-4 (PMC8709855; doi:10.1038/s41598-021-04047-4)
Supplement: Supplementary file 1 — Supplementary Information. [file 41598_2021_4047_MOESM1_ESM.docx]

Supporting Information

**Reporter Gene-Based Optoacoustic Imaging of *E. coli* Targeted Colon Cancer *In vivo***

*Misun Yun^1^, Sung-Hwan You^2^, Vu Hong Nguyen^3^, Jaya Prakash^1,4^, Sarah Glasl^1^, Vipul Gujrati ^1,6^, Hyon E. Choy^5^, Andre C. Stiel^1^, Jung-Joon Min^2*^ and Vasilis Ntziachristos^1,6*^*

^1^ Institute of Biological and Medical Imaging, Helmholtz Zentrum München GmbH, Neuherberg 85764, Germany.

^2^ Department of Nuclear Medicine, Chonnam National University Medical School, Gwangju, Republic of Korea

^3^ Department of Experimental Therapeutics, Beckman Research Institute of City of Hope, Duarte, California, USA

^4^ Department of Instrumentation and Applied Physics, Indian Institute of Science, C. V. Raman Avenue, Bengaluru- 560 012, India

^5^ Department of Microbiology, Chonnam National University Medical School, Gwangju, Republic of Korea

^6^ Chair of Biological Imaging, Center for Translational Cancer Research (TranslaTUM), School of Medicine, Technical University of Munich, Munich 81675, Germany.

Correspondence: [bioimaging.translatum@tum.de](mailto:bioimaging.translatum@tum.de) (V.N.), [jjmin@jnu.ac.kr](mailto:jjmin@jnu.ac.kr) (J.J.M.)

**Keywords:** Cancer imaging, *E. coli*, Melanin, Optoacoustic (OA) imaging, Colon cancer.


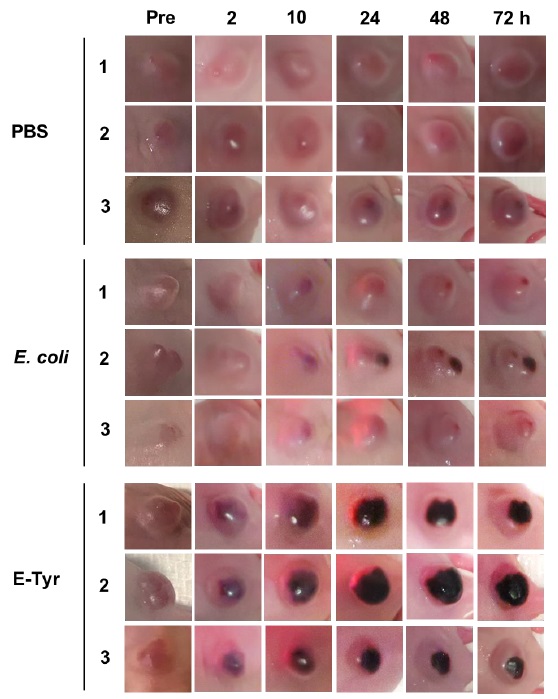


**Figure S1.** Photographs of representative tumors in each group were taken before (pre) and after injection (indicated times).


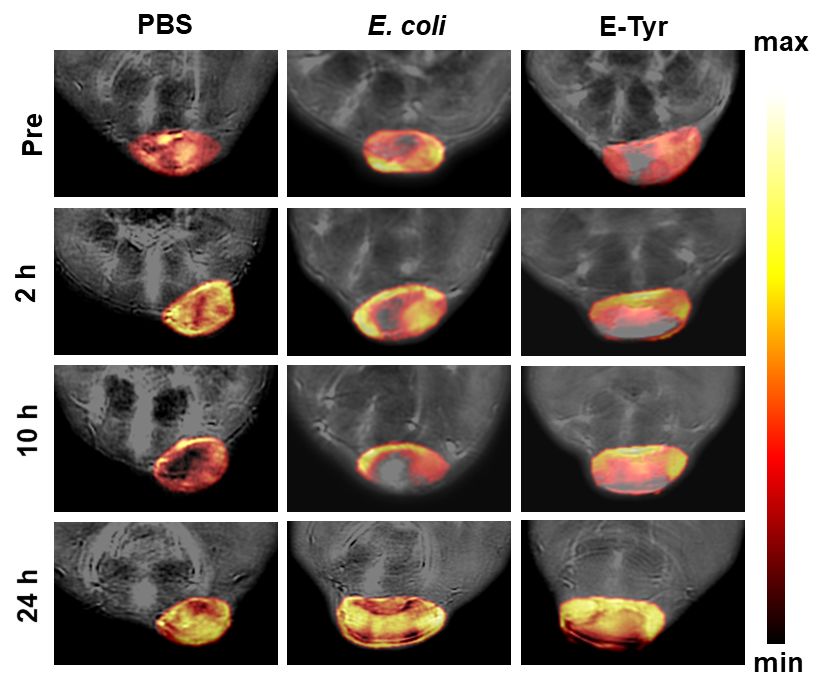


**Figure S2.** Representative images of tumors showing the spatial distribution of oxygen saturation at different time intervals.

(A)

(B)

**
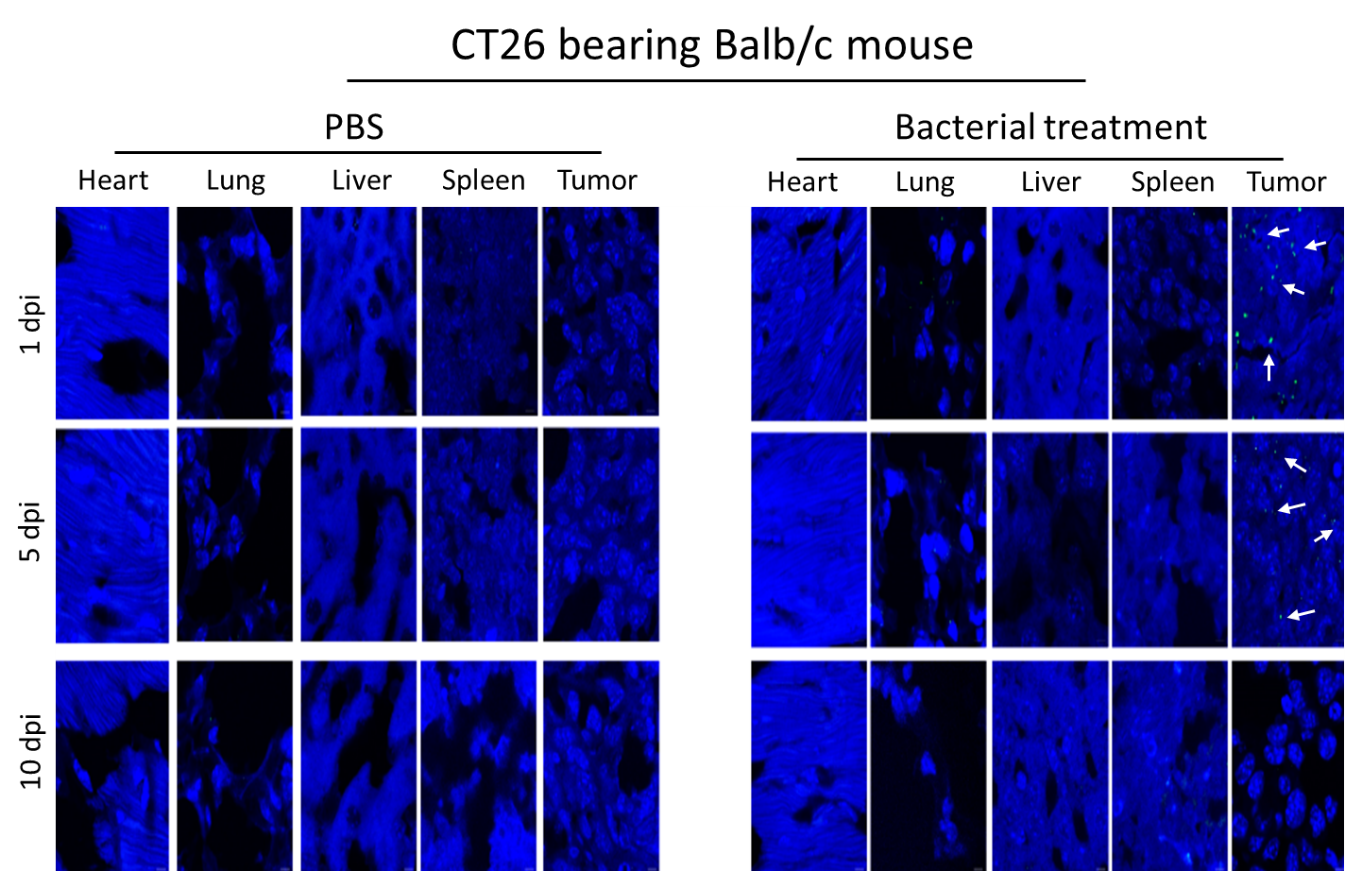
**

**Figure S3. Bacterial colonization in CT26 tumor-bearing Balb/c mice.** CT26 tumor-bearing competent mice received 10^8^ cfu of E-Tyr through intravenous injection. Heart, lung, liver, spleen, and tumor tissues were harvested for further experiments. **(A)** Viable bacterial cell number was counted and **(B)** immunofluorescence staining was performed by using an anti-E.coli antibody (green) and DAPI (4, 6-diamidino-2-phenylindole) (blue) for nuclei staining at the indicated time points (n=3 mice/group**).**

(A)

(B)


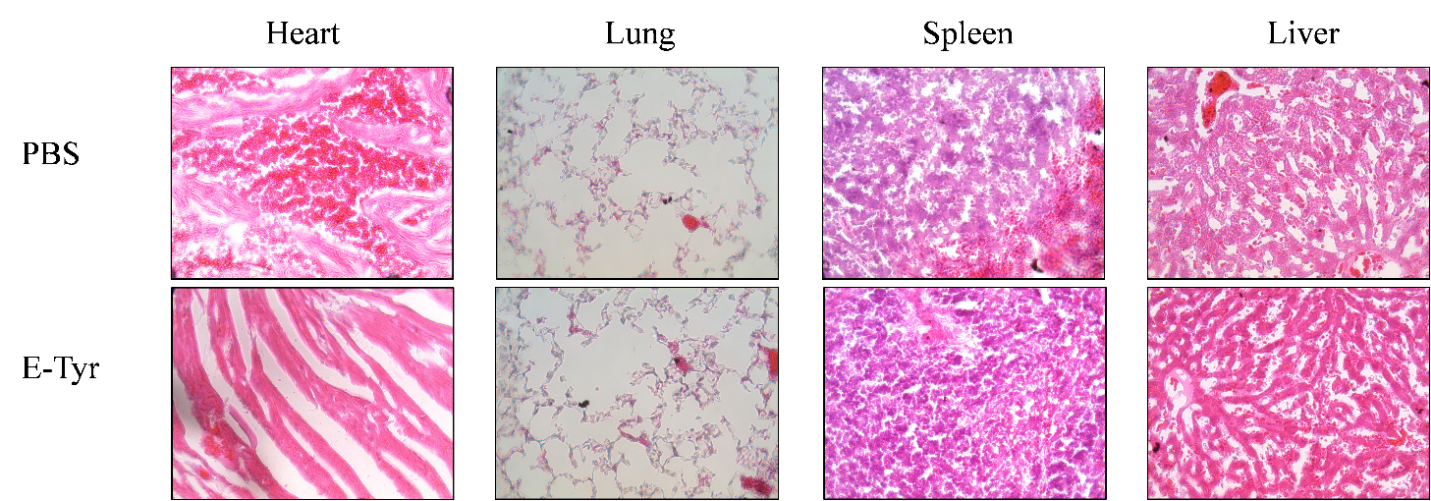


**Figure S4. Effect of E-tyr on survival and long-term toxicity of mice**. Competent Balb/c mice or immunocompromised Balb/c athymic nu-/nu- mice received 10^8^ cfu of E-Tyr/mouse through intravenous injection (n=3 per group). **(A)** Survival of bacteria-infected mice as monitored for over one month and **(B)** H&E staining (40X) of normal tissue sections at day 5 after bacterial injection.
